# Effect of LaCoO_3_ Synthesized via Solid-State Method on the Hydrogen Storage Properties of MgH_2_

**DOI:** 10.3390/ma16062449

**Published:** 2023-03-19

**Authors:** Noratiqah Sazelee, Muhamad Faiz Md Din, Mohammad Ismail, Sami-Ullah Rather, Hisham S. Bamufleh, Hesham Alhumade, Aqeel Ahmad Taimoor, Usman Saeed

**Affiliations:** 1 Energy Storage Research Group, Faculty of Ocean Engineering Technology and Informatics, University Malaysia Terengganu, Kuala Terengganu 21030, Malaysia; atiqahsazelee19@gmail.com; 2 Department of Electrical and Electronic Engineering, Faculty of Engineering, National Defence University of Malaysia, Kem Sungai Besi, Kuala Lumpur 57000, Malaysia; faizmd@upnm.edu.my; 3 Department of Chemical and Materials Engineering, King Abdulaziz University, P.O. Box 80204, Jeddah 21589, Saudi Arabia; rathersami@kau.edu.sa (S.-U.R.); hbamufleh@kau.edu.sa (H.S.B.); halhumade@kau.edu.sa (H.A.); ataimoor@kau.edu.sa (A.A.T.); umsaeed@kau.edu.sa (U.S.)

**Keywords:** cobalt lanthanum oxide, magnesium hydride, metal oxide, solid-state hydrogen storage

## Abstract

One of the ideal energy carriers for the future is hydrogen. It has a high energy density and is a source of clean energy. A crucial step in the development of the hydrogen economy is the safety and affordable storage of a large amount of hydrogen. Thus, owing to its large storage capacity, good reversibility, and low cost, Magnesium hydride (MgH_2_) was taken into consideration. Unfortunately, MgH_2_ has a high desorption temperature and slow ab/desorption kinetics. Using the ball milling technique, adding cobalt lanthanum oxide (LaCoO_3_) to MgH_2_ improves its hydrogen storage performance. The results show that adding 10 wt.% LaCoO_3_ relatively lowers the starting hydrogen release, compared with pure MgH_2_ and milled MgH_2_. On the other hand, faster ab/desorption after the introduction of 10 wt.% LaCoO_3_ could be observed when compared with milled MgH_2_ under the same circumstances. Besides this, the apparent activation energy for MgH_2_–10 wt.% LaCoO_3_ was greatly reduced when compared with that of milled MgH_2_. From the X-ray diffraction analysis, it could be shown that in-situ forms of MgO, CoO, and La_2_O_3,_ produced from the reactions between MgH_2_ and LaCoO_3_, play a vital role in enhancing the properties of hydrogen storage of MgH_2_.

## 1. Introduction

Hydrogen is increasingly seen as an energy carrier owing to its non-toxicity, abundant resources, positive environmental impact, and high energy density [1,2,3]. Nowadays, searching for effective hydrogen storage technologies is commonly recognized as one of the major difficulties faced by the hydrogen economy [4,5]. Solid-state hydrogen storage materials have drawn a significant amount of interest because of their safety consideration, cheapness, and high gravimetric capacity [6]. Over the past decade, MgH_2_ gained research interest due to its outstanding reversibility, low cost, non-toxicity, an abundance of resources, and high gravimetric hydrogen capacity (7.60 wt.%) [7,8,9]. Fortunately, practical applications of MgH_2_ are severely restricted by the slow reaction kinetics and high dissociation temperature [10,11,12]. Recently, significant improvements have been made by producing nanocrystalline MgH_2_ powders by the addition of metal oxide additives such as Nb_2_O_5_ [13], TiO_2_ [14], CoTiO_3_ [15], CoMoO_4_ [16], and MnMoO_4_ [17], through ball milling method to enhance hydrogen storage performance of MgH_2_. Rare earth metals are considered as one of the most intriguing additives/catalysts used in solid-state materials. For example, Ismail [18] found that after the addition of 10 wt.% LaCl_3_ into MgH_2_, hydrogen started to be released at 300 °C, 50 °C lower than with milled MgH_2_. It is revealed that the formation of MgCl_2_ and Mg–La alloy during the heating process of the composites gives a vital role in enhancing the performance of hydrogen storage of MgH_2_. In our previous study [19], adding 10 wt.% LaFeO_3_ to MgH_2_ positively affected the hydrogen sorption properties of MgH_2_. Compared with pure MgH_2_, the introduction of 10 wt.% of LaFeO_3_ reduced the desorption temperature by 120 °C. Further studies have exposed that ab/desorption kinetics of MgH_2_ were improved by the formation of Fe, MgO, and La_2_O_3_ phases during the heating process. For instance, Soni et al. [20] introduced LaF_3_ into MgH_2_ and proved that the samples started to release hydrogen at 320 °C, 40 °C lower than with milled MgH_2_. In addition, milled MgH_2_ absorbed only 2.00 wt.% hydrogen in 2.5 min, while MgH_2_ + LaF_3_ could absorb 4.90 wt.% of H_2_ under the same circumstances. Wu et al. [21] reported that adding LaNiO_3_ significantly enhanced the desorption and absorption kinetics of MgH_2_. Further investigation revealed that in situ formations of Mg_2_NiH_4_ and LaH_3_ have a synergistic effect that can serve as a “hydrogen pump”, hence enhancing the sorption kinetics of MgH_2_. The research led by Zhang and co-workers [22] discovered that after introducing LaNi_4.5_Mn_0.5_ to MgH_2_, excellent catalytic activity was observed. Interestingly, at 300 °C, the composites could desorb 6.60 wt.% of H_2_ in less than 360 s.

Besides this, according to Juahir et al. [23], doping MgH_2_ with Co_2_NiO lowered the starting hydrogen release and enhanced the ab/desorption kinetics of MgH_2_. According to their research, the formation of Co_1.29_Ni_1.71_O_4_ and Mg–Co alloy served as a real catalyst in enhancing the hydrogen sorption properties of MgH_2_. In comparison to other ferrites (ZnFe_2_O_4_, CoFe_2_O_4_, MnFe_2_O_4_, and Mn_0.5_Zn_0.5_O_4_), Zhang et al. [24] came to the finding that CoFe_2_O_4_ had the best catalytic performance in enhancing the hydrogen storage performance of MgH_2_. Furthermore, Cabo et al. [25] discovered that the addition of Co_3_O_4_ and NiCo_2_O_4_ additives decreased the starting desorption temperature of MgH_2_. In particular, Mandzhukova et al. [26] analyzed the effect of NiCo_2_O_4_ on the kinetic performance of the Mg/MgH_2_ system, and revealed that the kinetic properties of Mg were drastically enhanced. Liu et al. [27] used a reduction reaction method to synthesize Co@CNT and found that the doped samples began to release hydrogen at 324 °C, which was lower than that of the bulk samples (420 °C). Further research indicated that the energy barrier for hydrogen dissociation can be substantially reduced by Co and Co(II).

Motivated by previous research, two promising materials (La and Co) clearly demonstrate that LaCoO_3_ improves hydrogen ab/desorption kinetics and lowers the MgH_2_ desorption temperature. In this paper, different amounts of LaCoO_3_ were milled together to make MgH_2_–x wt.% LaCoO_3_ (where x is 5, 10, 15, and 20) composites. LaCoO_3_ was used as an additive to see how this material affected the hydrogen sorption properties of MgH_2_. To date, this is the first research into the hydrogen storage performance of MgH_2_/LaCoO_3_ composites for solid-state materials.

## 2. Materials and Methods

The LaCoO_3_ material was synthesized by using citric acid (≥98% pure; Sigma Aldrich, St. Louis, MO, USA), lanthanum oxide (≥99.9% pure; Aldrich Chemical Compound, Milwaukee, WI, USA), and pure cobalt oxide (99.99% pure; Sigma Aldrich, St. Louis, MO, USA) as starting materials with 0.121 g, 0.081 g, and 0.040 g respectively. The powders were mixed and thoroughly ground in an agate mortar. The solid mixture was then placed into crucibles made of alumina and calcined at 950 °C in a furnace for 5 h. In a planetary ball mill (NQM-0.4), using stainless steel vials and 4 balls at 400 rpm with a 40:1 ball-to-powder ratio, the various weight percentage (5, 10, 15, and 20) of LaCoO_3_ were milled together with MgH_2_ (≥95% pure; Sigma Aldrich, St. Louis, MO, USA). This milling approach was carried out for 1 h in different directions (milling = 15 min, rest = 2 min, mill again = 15 min). In an argon atmosphere, the MBRAUN UNIlab glove box was used for all the preparations (including weighing).

Sievert-type pressure composition temperature (Advanced Materials Corporation, Pittsburgh, PA, USA) was used to investigate the temperature-programmed-desorption (TPD) and hydrogen ab/desorption kinetics for all of the samples. Approximately, 400 mg of the samples were used in each test. All the samples were heated to 450 °C from ambient temperature for the TPD analyses at a rate of 5 °C/min. The absorption kinetics were carried out at 250 °C (33.0 atm), meanwhile, the desorption kinetics were evaluated at 300 °C (1.0 atm). To observe the hydrogen desorption behavior of MgH_2_, differential scanning calorimetry (DSC) was performed on a Mettler Toledo TGA/DSC 1. The samples were heated from 30 to 500 °C at rates of 15, 20, 25, and 30 °C/min under constant argon flow (50 mL). An alumina crucible in a glove box was filled with about 3–5 mg, and to prevent oxidation, the samples were then placed in a sealed glass bottle. To scrutinize the phase structure of the samples, X-ray diffraction (XRD) spectra were recorded in the range of 20°–80° using Cu-Kα radiation to analyze the phase structure of each sample. Scan speeds of 2.00°/min were used for θ–2θ scans. Prior to this, a small portion of the samples was evenly distributed on a sample holder and sealed with scotch tape to prevent oxidation.

The morphology of the samples was examined using scanning electron microscopy (SEM; JEOL, Akishima, Tokyo, Japan) (JSM-6360LA). In a vacuum state, the gold spray was applied to the samples after being prepared on carbon tape. Moreover, to further examine the sample’s chemical bond, a Shimadzu IRTracer-100, Kyoto, Japan Fourier Transform Infrared spectroscopy was used. Attenuated total reflectance (ATR) was used to measure the spectra at room temperature for 40 scans, between 2000 and 400 cm^−1^, with a resolution of 4 cm^−1^. At room temperature, Raman spectroscopy was performed using Renishaw Raman spectroscopy (532 nm radiation) extended with 0.1% power laser measurement.

## 3. Results and Discussion

**Figure 1:**
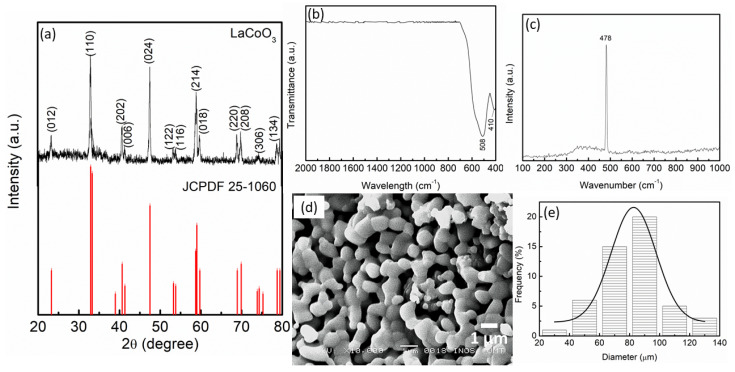
(**a**) XRD pattern, (**b**) FTIR spectra, (**c**) Raman spectra, (**d**) SEM images, and (**e**) PSD of LaCoO_3_.

Calcining the samples at 950 °C for 5 h yielded well-crystallized pure LaCoO_3_ (JCPDF: 25-1060), in the rhombohedral structure as shown in Figure 1a. All diffraction lines corresponding to (012), (110), (202), (006), (024), (122), (116), (214), (018), (220), (208), (306), and (134) planes were closely matched to 23.25°, 32.89°, 40.64°, 41.32°, 47.49°, 53.24°, 53.78°, 58.79°, 59.73°, 68.93°, 69.90°, 74.32°, and 78.74°, respectively, and were all closely matched to the previously reported data [28,29]. The crystallite sizes (L) were estimated at 20.85 nm, through the Scherrer formula as shown in Equation (1) below:

L = Kλ/*β* cos θ (1)

where shape factor K = 0.94 constant, λ = X-ray used (0.154 nm), *β* (physical broadening) = full width at half the maximum, and θ = angle of Bragg’s diffraction. The FTIR transmission of LaCoO_3_ is indicated in Figure 1b. A peak at 508 cm^−1^ is ascribed to the Co–O bond as reported by Sarker and Razzaque [28], and Worayingyong et al. [30]. The La–O bond as confirmed by Radev et al. [31], corresponds to the peak at 410 cm^−1^. The Raman spectra shown in Figure 1c illustrates typical characteristics of LaCoO_3_ at 478 cm^−1^, confirming the formation of La–O [32]. It can be evidenced that the pure LaCoO_3_ was successfully synthesized by using the solid-state method based on the results of XRD, FTIR, and Raman spectroscopy. Meanwhile, Figure 1d shows the morphology of LaCoO_3_, where the agglomeration of particles of different sizes can be seen as indicated in a previous study [33]. Besides this, the particle sizes distribution (PSD) of the LaCoO_3_ particle was calculated by using ImageJ (version 2022). Based on Figure 1e below, the PSD of LaCoO_3_ was 82.71 µm.

**Figure 2:**
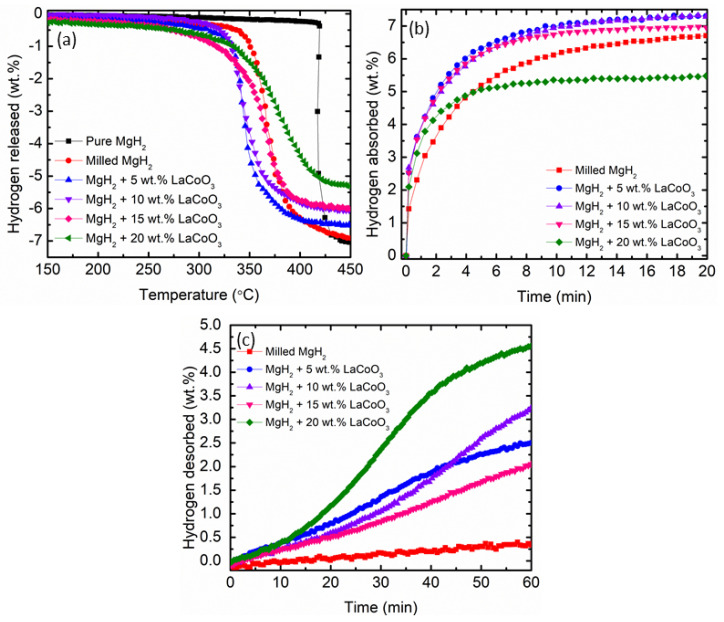
(**a**) Temperature-programmed desorption, (**b**) Isothermal absorption kinetics at 250 °C, and (**c**) Isothermal desorption kinetics at 300 °C.

The impact of LaCoO_3_ on the desorption temperature of MgH_2_ was measured using the TPD profile of gas desorption from the samples, as displayed in Figure 2a. Pure MgH_2_ and milled MgH_2_ both had onset desorption temperatures of 420 °C and 350 °C, respectively. It was discovered that the milling process had an impact on the decomposition of MgH_2_. According to Sokano et al. [34], milled MgH_2_ has a lower onset desorption temperature, which is 328 °C, compared with that of pure MgH_2_ (418 °C). However, the onset desorption temperature was shifted from 350 °C to a starting temperature below 325 °C when different weight percent of LaCoO_3_ were added to MgH_2_. The onset desorption temperature of 5, 10, 15, and 20 wt.% of LaCoO_3_ with MgH_2_ was 316, 322, 310, and 323 °C, respectively. Meanwhile, the desorption capacity of 5, 10, 15, and 20 wt.% of LaCoO_3_ with MgH_2_ was 6.57, 6.06, 6.03, and 5.30 wt.%, respectively. A study led by Pandey et al. [35] proved that adding TiO_2_ to MgH_2_ lowered the onset desorption temperature to 335 °C, 55 °C lower than that of pure MgH_2_. Despite the fact that adding LaCoO_3_ lowered the desorption temperature of MgH_2_, the hydrogen desorption capacity of xwt.% of LaCoO_3_ (where x is 5, 10, 15, and 20 wt.%) with MgH_2_ decreased due to the dead weight of LaCoO_3_.

The isothermal absorption measurement of the milled MgH_2_ and xwt.% of LaCoO_3_ (where x is 5, 10, 15, and 20 wt.%) with MgH_2_ was further conducted under 33.0 atm at 250 °C, as depicted in Figure 2b. The result proved that adding 5, 10, 15, and 20 wt.% of LaCoO_3_ with MgH_2_ could absorb 7.30, 7.30, 6.99, and 5.49 wt.%, respectively, within 20 min. Meanwhile, milled MgH_2_ could only absorb 6.68 wt.% under the same circumstances. The amount of hydrogen absorption for 20 wt.% of LaCoO_3_ with MgH_2_ was lower by 1.19 wt.%, compared with that of milled MgH_2_. This was due to the possibility that too much additive in the composite may block the diffusion path of hydrogen [36]. A previous study reported by Sulaiman et al. [37] indicated that the amount of Na_3_FeF_6_ affects the hydrogen absorption behavior of MgH_2_. The addition of excess Na_3_FeF_6_ catalyst into MgH_2_ obstructs the hydrogen diffusion by blocking the diffusion path, which limits the Mg-H reaction. However, faster absorption kinetics of MgH_2_ could be seen within 4 min after the addition of 20 wt.% LaCoO_3_. As evidenced by the above experimental results, the hydrogen absorption kinetics of MgH_2_ can be improved by the presence of LaCoO_3_.

To compare hydrogen desorption properties of different weight percentages of LaCoO_3_ with MgH_2_ and milled MgH_2_, an isothermal desorption test was conducted at 300 °C for 1 h, as presented in Figure 2c. It is clear that MgH_2_–LaCoO_3_ composites demonstrated faster desorption kinetics than milled MgH_2_. An amount of 5 wt.% of LaCoO_3_ with MgH_2_, and 10 wt.% of LaCoO_3_ with MgH_2_ released H_2_ at approximately 2.46 and 3.24 wt.%, respectively. In addition, 15 wt.% of LaCoO_3_ with MgH_2,_ and 20 wt.% of LaCoO_3_ with MgH_2_ desorbed 2.01 wt.% and 4.53 wt.% of H_2_, respectively. However, milled MgH_2_ only released 0.34 wt.% of H_2_ under the same circumstances. Table 1 summarizes the onset desorption temperature, the capacity of absorption kinetics at 250 °C, and desorption kinetics at 300 °C for pure MgH_2_, milled MgH_2_, and composites of different LaCoO_3_ weight percentages with MgH_2_. Considering the onset desorption temperature, absorption and desorption kinetics of each sample, 10 wt.% of LaCoO_3_ with MgH_2_ composites were chosen for further investigation.

To obtain a greater understanding of the kinetic mechanism in hydrogen storage materials, kinetic models were used to describe absorption and desorption behaviors. In this study, the kinetic mechanism was investigated by using the Johnson-Mehl-Avrami (JMA) and Contracting Volume (CV) equations as can be seen in Table 2 [38].

**Figure 3:**
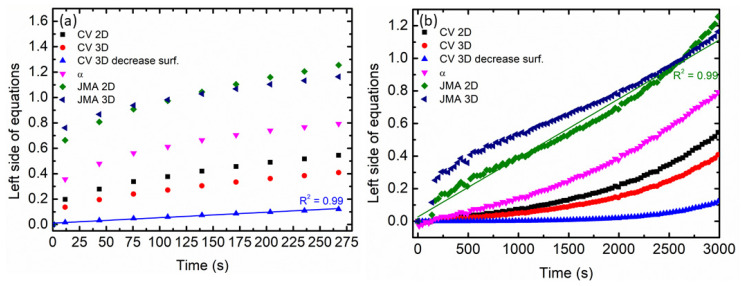
The resulting calculation of different kinetic equations is described in Table 2 for (**a**) absorption kinetics at 250 °C and (**b**) desorption kinetics at 300 °C of 10 wt.% of LaCoO_3_ doped MgH_2_.

The absorption and desorption kinetic curves for 10 wt.% LaCoO_3_ with MgH_2_ composites are illustrated in Figure 3a,b below. The kinetic curves for the composites were calculated for the reacted fraction in the range from 0 to 80%. Based on the figure below, the absorption process at 250 °C can be best described by the CV 3D decrease surface while the desorption process at 300 °C can be best described by the JMA 2D.

**Figure 4:**
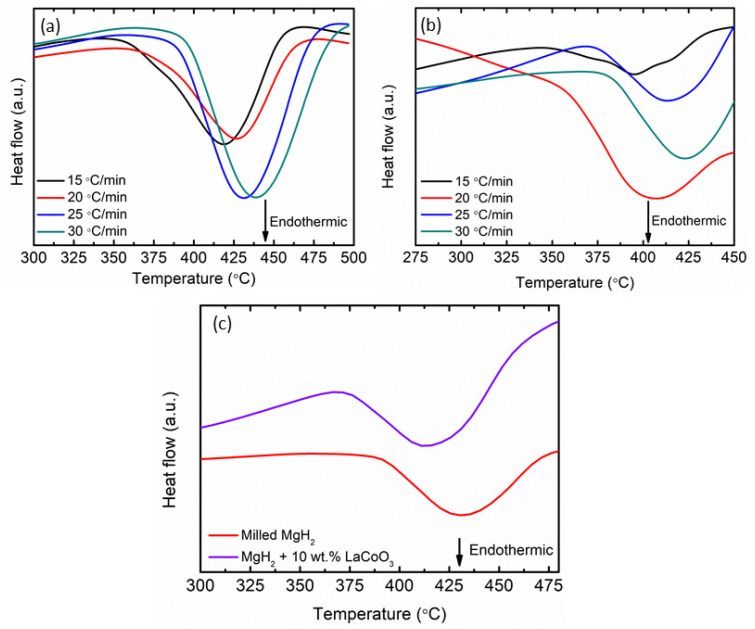
DSC curves of (**a**) milled MgH_2_ at 15, 20, 25, and 30 °C/min, (**b**) 10 wt.% of LaCoO_3_ with MgH_2_ at 15, 20, 25, and 30 °C/min, and (**c**) milled MgH_2_ and 10 wt.% of LaCoO_3_ with MgH_2_ at 25 °C/min.

DSC analyses were used to look into the effect of LaCoO_3_ on the desorption kinetics of MgH_2_. Figure 4a exhibits the DSC curves of milled MgH_2_, while Figure 4b indicates the 10 wt.% of LaCoO_3_ with MgH_2_ composites heated at various heating rates. As the heating rises, the hydrogen desorption peaks move to a higher temperature. An endothermic peak for both samples was detected in Figure 4c for a heating rate of 25 °C/min, revealing that the decomposition from MgH_2_ to Mg had occurred. As indicated in Figure 4c, the endothermic peak for milled MgH_2_ was 433 °C, while the temperature was shifted to a lower temperature after 10 wt.% of LaCoO_3_ was added (415 °C). From the results obtained for 10 wt.% of LaCoO_3_ with MgH_2_, the desorption peak temperature by DSC and TPD were 415 °C and 322 °C, respectively. This was due to the different atmospheres and heating rates as explained in our previous research [39,40]. A similar outcome had been observed by Verma et al. [41], which revealed that different desorption temperatures for DSC and TPD could be detected when the experiment was conducted under different circumstances.

The Kissinger method was used for both samples to evaluate the effect of LaCoO_3_ additives on desorption apparent activation energy (*E_A_*), as shown in Equation (2) below

**Figure 5:**
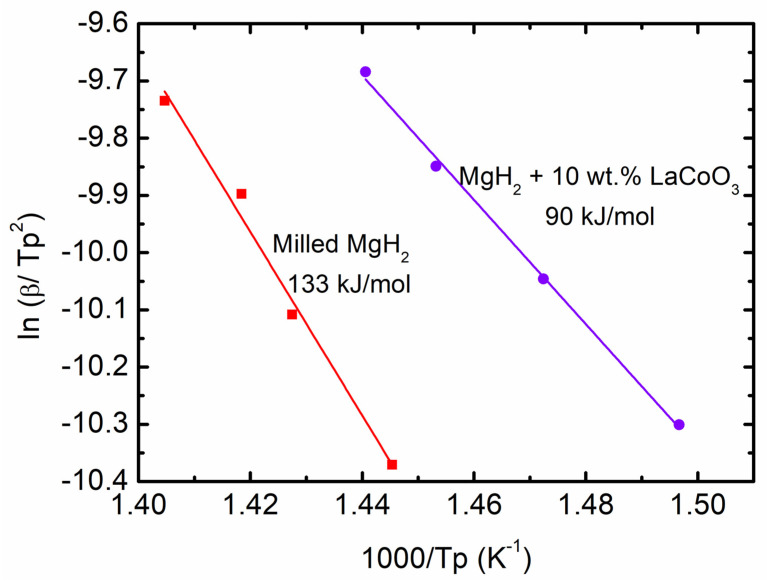
Activation energy of milled MgH_2_ and 10 wt.% of LaCoO_3_ with MgH_2_.

In [*β*/*T_p_*^2^] = −*E_A_*/R*T_p_* + A (2)

where A = linear constant, *T_p_* = peak temperature in the DSC curve, R = gas constant, and *β* = heating rate of the samples. Hence, the *E_A_* of the thermal decomposition for 10 wt.% of LaCoO_3_ with MgH_2_ based on Equation (2) was approximately 90 kJ/mol, as demonstrated in Figure 5. Conversely, the *E_A_* for milled MgH_2_ was only 133 kJ/mol. The *E_A_* of 10 wt.% of LaCoO_3_ with MgH_2_ was lower than those of the other additives from previous studies, such as MgH_2_–KNbO_3_ [42], MgH_2_–Co@C [43], and MgH_2_–SrFe_12_O_19_ [6]. According to the findings, overcoming the barrier for converting MgH_2_ into Mg requires an *E_A_* of 90 kJ/mol, in the presence of 10 wt.% LaCoO_3_. It is also worth noting that LaCoO_3_ additives lower the desorption peak temperature during the desorption processes of MgH_2_.

**Figure 6:**
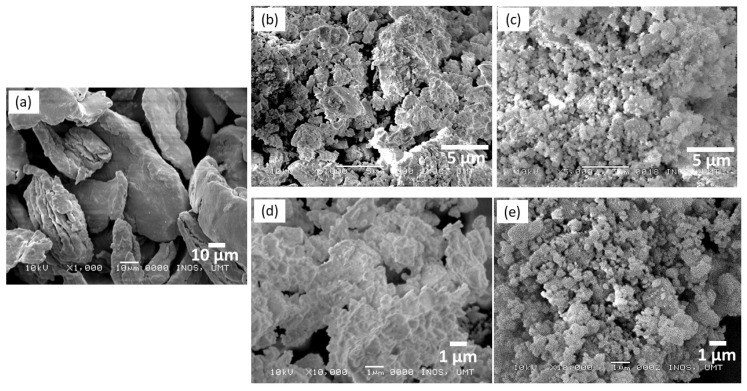
SEM images of (**a**) pure MgH_2_, (**b**) milled MgH_2_ at 5000× magnification, (**c**) 10 wt.% of LaCoO_3_ with MgH_2_ at 5000× magnification, (**d**) milled MgH_2_ at 10,000× magnification, and (**e**) 10 wt.% of LaCoO_3_ with MgH_2_ at 10,000× magnification.

The morphologies of pure MgH_2_, milled MgH_2_, and 10 wt.% of LaCoO_3_ with MgH_2_ were investigated by SEM as shown in Figure 6. The SEM of pure MgH_2_ shown in Figure 6a revealed that the particles have a larger size and flakelike shapes, as reported by Czujko et al. [44]. Even though the samples were analyzed at different magnifications, as displayed in Figure 6b,d, both samples suggest that ball milling produces inhomogeneity, some agglomeration, and reduction in the MgH_2_ samples caused by the ball collision. Smaller particle sizes for the milled MgH_2_ suggests an enhancement in the desorption temperature of MgH_2_. Besides, Shang and colleagues [45] indicated that the particle sizes in the submicron range can be achieved by milling Mg with or without the presence of additives. Compared with milled MgH_2_ samples, it was obvious that particles the size of 10 wt.% of LaCoO_3_ with MgH_2_, as illustrated in Figure 6c,e, became smaller and less agglomerated, which may accelerate the desorption and absorption kinetics of MgH_2_ due to the increase of specific surface area, even when the samples were investigated at different magnifications. Chawla et al. [46] exposed that mechanical milling of Mg with PdCl_2_ increases the surface area, resulting in a reduction in hydrogen atom diffusion length and an improvement in hydrogen ab/desorption kinetics. A similar outcome was also reported by Zinsou and co-workers [47] which also revealed that smaller particles are expected to release hydrogen at a lower temperature than samples that have larger particle sizes.

**Figure 7:**
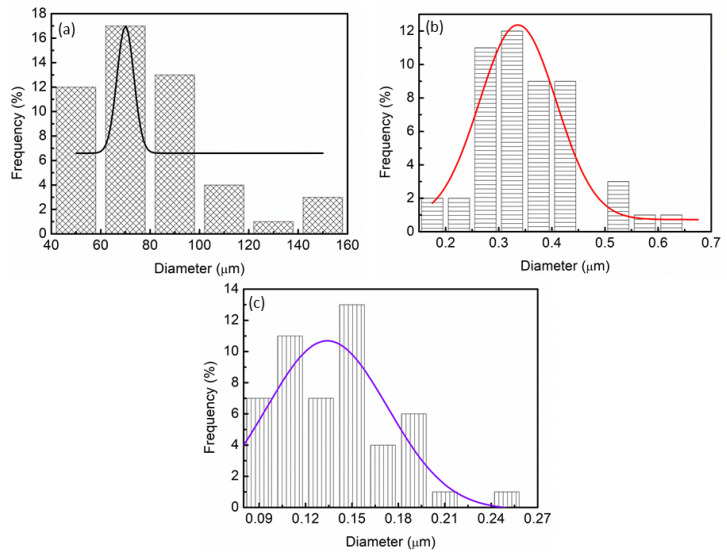
The PSD curves (**a**) pure MgH_2_, (**b**) milled MgH_2_, and (**c**) 10 wt.% of LaCoO_3_ with MgH_2_.

Particle size distribution was calculated by using Image J. The particle size distribution for pure MgH_2_ is generally known to be 70 µm, as displayed in Figure 7a. Meanwhile, based on Figure 7b, the particle size distribution of MgH_2_ started to decrease after MgH_2_ was milled for 1 h (approximately 0.34 µm). Xiao and colleagues [48] have described that the particle size of commercial MgH_2_ significantly reduced to ~300 nm after MgH_2_ was milled. However, it is safer to assume that longer milling times would not result in a reduction in particle size as revealed by Rahmaninasab et al. [49]. Rahmaninasab et al. [49] also reported that the particle size of MgH_2_ increased to 372 nm after the samples were milled for 40 h. Moreover, the particles of 10 wt.% of LaCoO_3_ with MgH_2_ were similar in size and less agglomerated, compared with those of milled MgH_2_, and most of the particles were single particles with 0.13 µm for particles size distribution, as indicated in Figure 7c. The addition of additive and milling methods effectively alters the distribution of MgH_2_. According to Si et al. [50], the increase in the small particles size was caused by the addition of Ni particles, which potentially improved the hydrogen storage performance of MgH_2_. Therefore, adding 10 wt.% of LaCoO_3_ reduces the particles size and shortens the diffusion length of MgH_2_, and contributes in improving MgH_2_ performance as observed.

**Figure 8:**
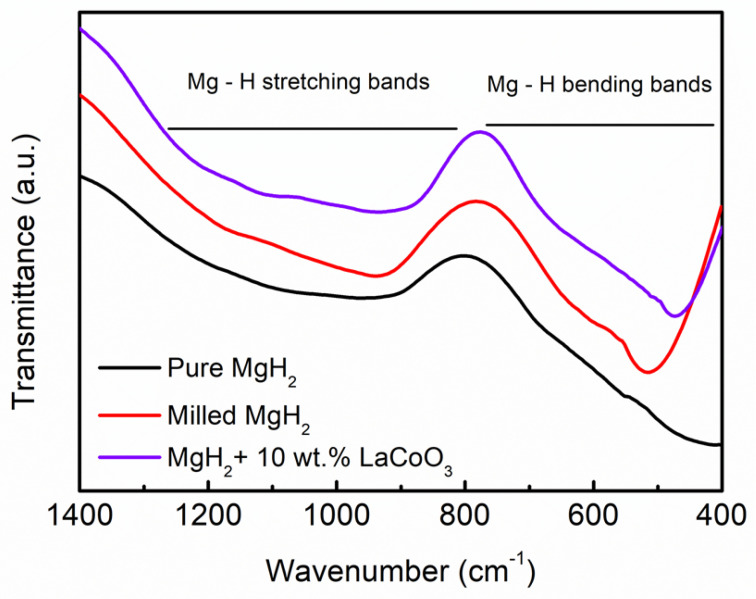
FTIR spectra of pure MgH_2_, milled MgH_2_, and 10 wt.% of LaCoO_3_ with MgH_2_.

Figure 8 presents the FTIR spectra of the samples before and after being doped with 10 wt.% LaCoO_3_. As seen from the figure below, the obvious signature bands for the Mg–H bending and Mg–H stretching are located between 400–800 cm^−1^ and 900–1200 cm^−1^, respectively, for pure MgH_2_, milled MgH_2_, and 10 wt.% of LaCoO_3_ with MgH_2_. The results suggest that no obvious reactions occurred due to its relatively low content of LaCoO_3_. Moreover, after adding 10 wt.% of LaCoO_3_ to MgH_2_, the bending bands of the samples tend to shift to lower wavenumbers, implying the weakness of the Mg–H bonds as proposed in a previous study [51].

**Figure 9:**
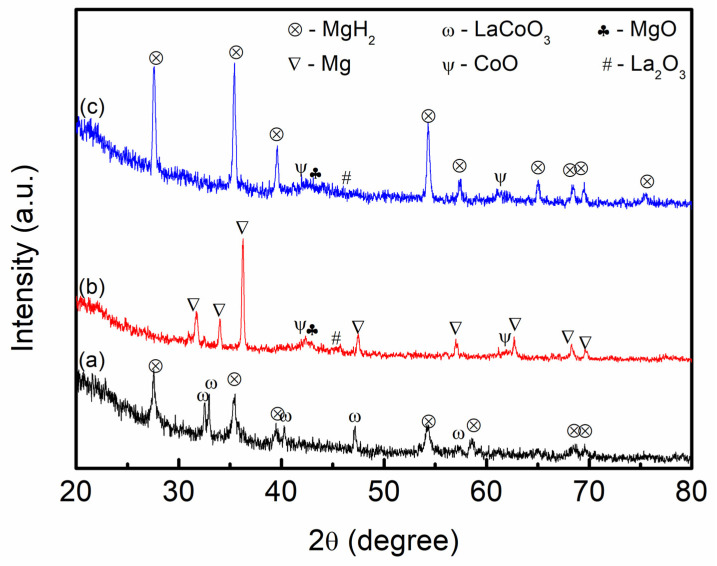
XRD pattern of 10 wt.% of LaCoO_3_ with MgH_2_ (a) milled for 1 h, (b) after desorption temperature at 450 °C, and (c) after absorption at 250 °C.

The XRD pattern shown here in Figure 9 was used to clarify the mechanism of 10 wt.% of LaCoO_3_ on MgH_2_ hydrogen storage performance. After 10 wt.% of LaCoO_3_ with MgH_2_ was milled for 1 h, as shown in Figure 9a, the peaks of the composites mainly corresponded to parent materials which are MgH_2_ and LaCoO_3_, indicating that LaCoO_3_ had not reacted with MgH_2_ during the milling process. Based on Figure 9b, after the composites were heated up to 450 °C, MgH_2_ peaks completely disappeared and transformed to Mg, implying that the decomposition process had occurred completely. A new peak of CoO, La_2_O_3_, and MgO was detected. The 10 wt.% of LaCoO_3_ with MgH_2_ during the absorption process at 250 °C had also been conducted, as shown in Figure 9c. The Mg peaks were converted to MgH_2_, revealing that the hydrogen absorption process had completely occurred. However, the peak of CoO, La_2_O_3_, and MgO were still detected. The following equation (Equation (3)) can be used to predict the reaction between 10 wt.% of LaCoO_3_ with MgH_2_:

MgH_2_ + 2LaCoO_3_ → 2CoO + La_2_O_3_ + MgO + H_2_ (3)

A prior study conducted by Rahman et al. [42] discovered that adding KNbO_3_ greatly reduced the onset desorption temperature from 370 °C to 327 °C, and lowered the *E_A_* by 61 kJ/mol, when compared with milled MgH_2_. Additionally, when as-synthesized Co@C was added to MgH_2_, the desorption temperature was reduced by 99 °C compared with milled MgH_2_ [43]. Furthermore, at 300 °C, MgH_2_-Co@C composites absorbed 5.96 wt.% of H_2_ in 10 min, and desorbed 5.74 wt.% of H_2_ in 1 h. Another metal oxide catalyst, SrFe_12_O_19_, also exhibited superior performance for MgH_2_ [6]. In comparison to milled MgH_2_, the introduction of SrFe_12_O_19_ decreased the *E_A_* and onset desorption temperature from 350 °C to 270 °C, and 133.31 kJ/mol to 114.22 kJ/mol, respectively. According to the findings, the in-situ formation of SrO, MgFe_2_O_4_, and Fe plays a crucial role in enhancing the hydrogen storage properties of MgH_2_. In this study, according to the results of the onset desorption temperature, ab/desorption kinetics, and the activation energy, adding the LaCoO_3_ (metal oxides) additive greatly enhances the hydrogen storage performance of MgH_2_. The onset desorption temperature was reduced by 28 °C and the *E_A_* was lowered by 43 kJ/mol. In addition, this composite could absorb 7.30 wt.% of H_2_ at 250 °C and desorb 3.24 wt.% of H_2_ at 300 °C, which is faster in kinetics compared with milled MgH_2_. The further study exposed that in situ-generated CoO, La_2_O_3_, and MgO could play a significant role in enhancing the dehydrogenation performance of MgH_2_.

According to Lee et al. [52], adding CoO has the best impact on the hydrogen sorption properties of Mg. Reactive grinding of Mg with Co/CoO causes defects and cracks in the surface of Mg particles, thus shortening diffusion distances between the samples. In addition, adding a minimal amount of CoO diminishes the dehydrogenation temperature and speeds up the dehydrogenation rate of LiBH_4_.NH_3_–3LiH system [53]. Furthermore, adding La_2_O_3_ to MgH_2_ improves the sorption properties [54]. Our previous study [19] also found that the introduction of 10 wt.% LaFeO_3_ positively affected the sorption kinetics of MgH_2_. After the addition of LaFeO_3_, the *E_A_* decreased by 32 kJ/mol. Besides this, when MgH_2_ was milled with MgO, the hydrogen kinetics were dramatically improved compared with MgH_2_ [55]. At 300 °C, faster hydrogen absorption and desorption kinetics can be accomplished in <100 s. Based on the discussions above, the introduction of LaCoO_3_ lowered the onset desorption temperature and enhanced the kinetic properties of MgH_2_ via the formation of in situ-generated CoO, La_2_O_3_, and MgO.

## 4. Conclusions

In conclusion, the different weight percentages of LaCoO_3_ have different effects on the hydrogen storage performance of MgH_2_. In comparison to milled and pure MgH_2_, the addition of LaCoO_3_ allows hydrogen to be released at a lower temperature. For 5 wt.%, 10 wt.%, 15 wt.%, and 20 wt.% of LaCoO_3_-doped MgH_2_ composites, the composites decomposed at 316 °C, 322 °C, 310 °C, and 323 °C, respectively, which were at lower temperatures than those of milled MgH_2_ and pure MgH_2_. In the reversibility evaluation, 5 wt.%, 10 wt.%, 15 wt.%, and 20 wt.% of LaCoO_3_-doped MgH_2_ samples absorbed 7.30 wt.%, 7.30 wt.%, 6.99 wt.%, and 5.49 wt.%, respectively, at 250 °C within 20 min. Meanwhile, milled MgH_2_ could absorb less than 6.68 wt.% of H_2_ under the same conditions. In addition, the isothermal desorption kinetics for 5 wt.%, 10 wt.%, 15 wt.%, and 20 wt.% of LaCoO_3_-doped MgH_2_ were 2.46 wt.%, 3.24 wt.%, 2.04 wt.%, and 4.53 wt.%, respectively, which were higher than that of milled MgH_2_ (0.34 wt.%). Through the DSC and Kissinger equation, the activation energy of 10 wt.% of LaCoO_3_ with MgH_2_ was 90 kJ/mol, which was 43 kJ/mol lower than that of milled MgH_2_. Furthermore, the doped samples present a smaller particle size compared with pure and milled MgH_2_, which allows more hydrogen to be absorbed/released. According to the XRD results, the in-situ formation of CoO, La_2_O_3_, and MgO plays a synergistic role in significantly improving MgH_2_ hydrogen storage performance.

## Figures and Tables

**Table 1:** Temperature-programmed desorption, absorption and desorption capacity of each sample.

Samples	Onset Desorption Temperature (°C)	Absorption Capacity (wt.%)	Desorption Capacity (wt.%)
Pure MgH_2_	420	-	-
Milled MgH_2_	350	6.68	0.34
5 wt.% LaCoO_3_ with MgH_2_	316	7.30	2.46
10 wt.% LaCoO_3_ with MgH_2_	322	7.30	3.24
15 wt.% LaCoO_3_ with MgH_2_	310	6.99	2.04
20 wt.% LaCoO_3_ with MgH_2_	323	5.49	4.53

**Table 2:** The equations for kinetic models used for absorption and desorption kinetics of this study.

Integrated Equation	Model
α = kt	Surface-controlled (chemisorption)
[−ln(1 − α)]^1/2^ = kt	JMA, n = 2 (e.g., two-dimensional growth of existing nuclei with constant interface velocity)
[−ln(1 − α)]^1/3^ = kt	JMA, n = 3 (e.g., two-dimensional growth of existing nuclei with constant interface velocity)
1 − (1 − α)^1/3^ = kt	CV 2D: contracting volume, three-dimensional growth with constant interface velocity
1 − (2α/3) − (1 − α)^2/3^ = kt	CV 3D: contracting volume, three-dimensional growth diffusion controlled with decreasing interface velocity

Where k = reaction rate constant, t = time, and α = reacted fraction.

## Data Availability

The data presented in this study are available on request from the corresponding author.
